# Immunofluorescence-Verified Sphingolipid Signatures Indicate Improved Prognosis in Liver Cancer Patients

**DOI:** 10.7150/jca.101330

**Published:** 2024-10-14

**Authors:** Lujuan Pan, Huijuan Huang, Pengpeng Zhang, Hua Li, Libai Lu, Mingwei Wei, Pin Zheng, Qi Wang, Junyu Guo, Yueqiu Qin

**Affiliations:** 1 Department of Gastroenterology, Affiliated Hospital of Youjiang Medical University for Nationalities, Baise, Guangxi, China.; 2 Guangxi Clinical Medical Research Center for Hepatobiliary Diseases, Guangxi, China.; 3 Department of General Surgery, Affiliated Hospital of Youjiang Medical University for Nationalities, Baise, Guangxi, China.; 4 Department of Lung Cancer Surgery, Tianjin Medical University Cancer Institute and Hospital, Tianjin, 300060, China.; 5 Department of Gastroenterology, Affiliated Hospital of Jiangsu University, Jiangsu University, Zhenjiang, China.

**Keywords:** Sphingolipid, Hepatocellular Carcinoma, Precision immunotherapy, Biomarkers, Immunofluorescence

## Abstract

**Background:** Hepatocellular carcinoma (HCC) is a highly heterogeneous malignancy, with its pathogenesis involving a complex interplay of molecular mechanisms, including cell cycle dysregulation, evasion of apoptosis, enhanced angiogenesis, and aberrant immune responses. Precision medicine approaches that target specific molecular subtypes through multi-omics integration hold promise for improving patient survival. Among the various molecular players, sphingolipids have emerged as pivotal regulators of tumor growth and apoptosis, positioning them as key targets in the search for novel anticancer therapies.

**Methods:** To identify critical genes involved in sphingolipid metabolism (SM), we employed the AUCell algorithm and correlation analysis in conjunction with scRNA-seq data. A robust prognostic risk model was developed using Cox proportional hazards and Lasso regression, and its predictive performance was validated using an independent cohort from the International Cancer Genome Consortium (ICGC). The model's evaluation also incorporated analyses of the tumor microenvironment (TME), immunotherapy responses, mutational landscape, and pathway enrichment across different risk strata. Finally, we conducted multiplex immunofluorescence assays to investigate the functional role of ZC3HAV1 in HCC.

**Results:** Our analysis yielded a 9-gene signature risk model with strong prognostic capabilities, effectively stratifying HCC patients into high- and low-risk groups, with significant differences in survival outcomes. Notably, the model revealed distinct variations in the immune microenvironment and responsiveness to immunotherapy between the risk groups. Further experimental validation identified ZC3HAV1 as a key gene, with multiplex immunofluorescence suggesting its involvement in promoting malignant progression in HCC through modulation of the epithelial-mesenchymal transition (EMT).

**Conclusion:** This sphingolipid metabolism-based prognostic model is not only predictive of survival in HCC but also indicative of immunotherapy efficacy in certain patient subsets. Our findings underscore the crucial role of sphingolipid metabolism in shaping the immune microenvironment, offering new avenues for targeted therapeutic interventions.

## 1. Introduction

Hepatocellular carcinoma (HCC) stands as a globally prioritized health concern, particularly pronounced in China and several Asian countries. HCC ranks as the third leading cause of cancer-related deaths worldwide, with annual incidence and mortality rates steadily on the rise^1^. By 2025, over one million individuals are projected to be impacted by primary liver cancer annually, posing a significant health challenge and societal burden 2. Hepatocellular carcinoma, constituting 90% of all cases, predominantly manifests as an invasive malignancy originating from liver cells 3, 4. Cholangiocarcinoma (CCA), the second major type of primary liver cancer, currently accounts for 10% of cases but is witnessing a rapid increase in incidence 5. Despite significant advances in clinical treatments for liver cancer over the past decades, surgical resection remains the sole curative option. However, a substantial number of liver cancer patients are diagnosed at an advanced stage where surgical resection is no longer viable, leading to cancer recurrence and metastasis even after early-stage liver cancer resection^6^, ^7^. Consequently, more effective approaches are needed to treat primary hepatocellular carcinoma and prevent its recurrence.

In cancer research, the investigation of tumor metabolism plays a pivotal role. Analysis of metabolites during carcinogenesis unveils the mechanisms, developmental processes, and therapeutic responses to cancer 8, 9. Firstly, the occurrence of cancer is often accompanied by aberrant expression of metabolites. Detection of these abnormal metabolites, particularly through metabolomics techniques, facilitates early detection and diagnosis of cancer 10. For instance, studies have identified significant differences in the metabolite composition of lung cancer patients compared to healthy individuals, providing novel biomarkers for early lung cancer diagnosis 11. Secondly, carcinogenesis is a multistep, multifactorial process, and an in-depth understanding of tumor metabolism aids in comprehending this process. Immune cells and immune-related molecules, collectively referred to as the tumor immune microenvironment, play crucial roles in both the tumor microenvironment and the development of liver cancer 12. Immunotherapy, hailed as the "Breakthrough of the Year" for cancer treatment in 2013, primarily involves activating or enhancing the body's immune system to recognize and attack tumor cells 13, 14. In liver cancer treatment, immunotherapy operates through immune checkpoint inhibitors (ICIs), cell immunotherapy, tumor vaccines, with ICIs, particularly, making notable breakthroughs in recent oncology treatments^15^. ICIs primarily function by blocking signaling pathways between tumor cells and immune cells, thereby relieving the inhibition of tumor cells by the immune system and reactivating immune cells to attack tumors, such as PD-1/PD-L1 inhibitors^16^.

The significance of tumor metabolism in cancer intervention and treatment, particularly with lipid dysregulation playing a pivotal role in liver cancer development, cannot be overstated^17^, ^18^. Sphingolipid metabolism, a critical subset of lipid metabolism, involves key molecules such as sphinganine, ceramides (Cer), sphingosine-1-phosphate (S1P), sphingomyelin, and glycosphingolipids. These components are essential constituents of cell membranes and organelles, integral to maintaining cellular homeostasis 19. Ceramides, central players in sphingolipid metabolism, mediate biological responses including growth inhibition, apoptosis, and senescence, whereas S1P promotes cell survival, proliferation, and tissue regeneration 20, 21. These two molecules exert diametrically opposing effects in tumorigenesis, underscoring the importance of maintaining a delicate balance between sphingolipids to preserve normal biological functions. Thus, sphingolipids, along with the enzymes and metabolites involved in their metabolism, hold potential as critical cancer biomarkers in disease progression 22-24.

Therefore, the aim of this study is to screen for Sphingolipid Metabolism-Related Genes (SMGs) in HCC and elaborate on their roles in the Tumor Microenvironment (TME) and HCC prognosis. Our newly developed SMGs model in this preliminary investigation demonstrates superior prognostic prediction for patients and guides immune therapy, potentially opening up new avenues for sphingolipid metabolism-oriented cancer treatment.

## 2. Materials and Methods

### 2.1 Acquisition and Integration of Transcriptomic Data

The RNA expression profiles, gene mutations, and relevant clinical data for liver cancer were retrieved from the TCGA database (n=1095). The dataset was split into a training group (70%) and a validation group (30%), where the training group was employed for model construction and the validation group for assessing model stability and accuracy. Additionally, liver cancer patient expression profiles from the ICGC were downloaded for external independent validation. All data were in TPM format, log2 transformed for subsequent analysis. The "SVA" package was utilized to adjust for data batch effects between TCGA and ICGC.

### 2.2 Acquisition and Analysis of scRNA-seq Data

The HCC single-cell dataset with the accession number GSE149614 containing 10 samples was obtained from the GEO database. Quality control for single-cell sequencing data was conducted using the "seurat" and "singleR" R packages. Filtering criteria included cells with mitochondrial gene content not exceeding 10%, cells with over 200 total genes, and genes expressed between 200 and 7000 at least in three cells to ensure high data quality. Linear regression models and "log normalization" methods were applied for further scaling and normalization of the remaining cells. The top 3000 highly variable genes were selected using the "FindVariableFeatures" function. To mitigate batch effects in downstream analysis arising from data originating from different samples, canonical correlation analysis (CCA) was employed using the "FindIntegrationIntegrators" function. Additionally, "IntegrateData" and "ScaleData" functions were used for comprehensive data integration and expansion. Principal Component Analysis (PCA) was conducted for dimensionality reduction to identify anchor points. The t-Distributed Stochastic Neighbor Embedding (t-SNE) method was employed to examine the top 20 PCs for meaningful clustering. Evaluation of cell cycle heterogeneity was performed based on cell cycle marker embedding from the "seurat" package.

### 2.3 Selection of Sphingolipid-Related Genes

The GeneCards database provided an extensive list of sphingolipid-related genes. We filtered out 110 SRGs with a correlation score greater than 1.0 for further in-depth investigation.

### 2.4 AUCell

Key genes most associated with sphingolipid metabolism activity were elucidated using scRNA-seq data. The "AUCell R" package facilitated the assignment of sphingolipid activity scores to each cell lineage based on the genomic activity state. The percentage of highly expressed gene sets in each cell was estimated by calculating the Area Under the Curve (AUC) values based on selected SRGs. The "AUCell explore holds" function identified cells actively involved in the sphingolipid genome. Cells were categorized into high and low sphingolipid-AUC groups based on median AUC scores, and visualization was achieved using the "ggplot2 R" tool.

### 2.5 Single-Sample Gene Set Enrichment Analysis (ssGSEA)

ssGSEA analysis was employed to precisely calculate gene set scores enriched in each sample and ascertain the Sphingolipid Metabolism (SM) score for each TCGA-HCC patient.

### 2.6 Construction of Sphingolipid-Related Risk Features

Initially, sphingolipid-related genes with prognostic value were extracted through univariate Cox analysis. Subsequently, Lasso regression was utilized for further screening of prognostic SRGs to construct a prognostic model. Risk scores were assigned to each HCC patient using this algorithm. Patients in the TCGA-HCC cohort were divided into high-risk and low-risk groups based on the median. Subsequent analysis of survival status changes between the two groups and assessment of the model's accuracy were performed.

### 2.7 Evaluation of Model Predictive Independence and Effectiveness

Risk scores, age, gender, pathological stage, and other clinical parameters were treated as independent prognostic factors. A column chart was designed to calculate 1-year, 3-year, and 5-year Overall Survival (OS) probabilities. To predict prognosis, Kaplan-Meier methods were used to plot survival curves, and log-rank tests were performed to assess statistical significance. Calibration curves and ROC curves were created to evaluate the accuracy of the nomogram. Decision curve analysis (DCA) was utilized to assess the net benefits of the nomogram and individual clinical features. Stratified analysis (including age, gender, clinical stage, and pathological T stage) was conducted to evaluate the prognostic significance of risk scores in clinical features.

### 2.8 Analysis of the Association Between the Prognostic Model and Tumor Immunity and Immunotherapy

Using the TIMER 2.0 database from the TCGA, we further investigated the degree of immune infiltration in HCC patients and obtained results encompassing seven evaluation methods. A heatmap was constructed to quantify the relative proportions of immune cells in the tumor microenvironment. Additionally, using the "GSEAB" R package, equipped with immune-related characteristics, gene sets in the prognostic risk assessment model underwent ssGSEA analysis. To evaluate and compare the relative frequencies of stromal cells, immune cells, and tumor cells in each risk group, the "estimate" R package was employed.

### 2.9 Creation of Mutation Maps and Investigation of Drug Sensitivity

Gene mutation data for HCC patients were extracted from the TCGA database, processed using the "maftools" package to generate gene mutation spectra for HCC patients. The obtained gene mutation data were integrated with risk scores. Furthermore, using the "pRRophetic" package in R software, median Inhibitory Concentrations (IC50) of various commonly used chemotherapy drugs were calculated to assess the correlation between risk scores and drug sensitivity. Finally, Wilcoxon signed-rank tests were conducted to compare IC50 differences between the two risk score groups.

### 2.10 Collection of Cells and Tissues for Cultivation

A retrospective collection was performed on 3 pairs of liver cancer patients who underwent tumor resection surgery and received ethical approval from the Affiliated Hospital of Youjiang Medical University for Nationalities. Tumor tissues (T) and precancerous tissues (N) were immediately placed in liquid nitrogen and stored at -80 ℃ for long-term preservation. Additionally, permission for subsequent experimental research was obtained from the hospital's Institutional Review Board.

### 2.11 Multiplex Immunofluorescence

Collected HCC tissue sections underwent steps such as deparaffinization and fixation for multiplex immunofluorescence staining to detect target antibody expression. Primary antibodies included ZC3HAV1 (rabbit, 1:100) and E-cadherin (mouse, 1:3000). Secondary antibodies were incubated with horseradish peroxidase. Multiple immunofluorescence images were analyzed using Caseviewer (C.V 2.3, C.V 2.0) and ZEN 3.3 (blue edition) image analysis software.

## 3. Results

### 3.1 Identification of Sphingolipid Metabolism-Related Cell Clusters

**Figure 1:**
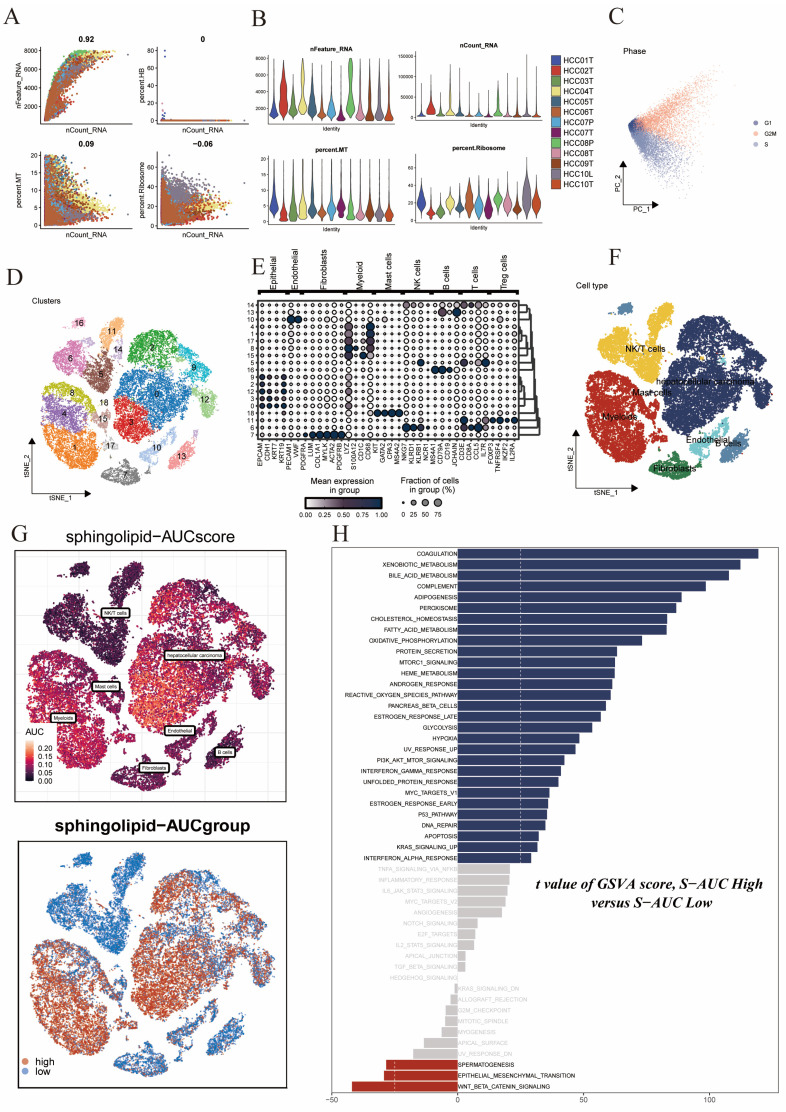
Sphingolipid Metabolism Characteristics in Single-Cell Data of Liver Cancer. (A-C) Quality control of single-cell data. (D) t-SNE plot showing different cell clusters in the single-cell dataset. (E) Bubble plot displaying typical marker genes for each cell cluster. (F) t-SNE plot indicating annotated cell types in the single-cell data of liver cancer (different colors represent different cell types). (G) AUCell scores of cell clusters. (H) GSVA differences among different AUCell score groups.

We obtained a public single-cell dataset from the GEO database for the identification of sphingolipid metabolism-related cell clusters. After a series of quality control procedures **(Figure 1A,B,C)**, such as restricting the percentages of mitochondrial genes, ribosomal genes, and red blood cell genes, and imposing requirements on sequencing depth, total intracellular sequences, and cell cycle, further analysis was conducted. Using dimensionality reduction algorithms, we classified all cells into 19 more detailed clusters **(Figure 1D)**, with marker genes for each cell cluster displayed in bubble plots** (Figure 1E)**. Overall, these cells mainly included fibroblasts, monocytes/macrophages, and tumor cells, among others** (Figure 1F)**. Subsequently, to elucidate the heterogeneity among these cell clusters, we utilized the AUCell R package to determine sphingolipid metabolism (SM) activity for each cell. We observed elevated AUC values in cell clusters such as hepatocellular carcinoma cells and enlarged cells **(Figure 1G)**. All cells were assigned AUC scores for respective SRGs, and based on the AUC score threshold, they were divided into two groups (high sphingolipid AUC group and low sphingolipid AUC group). Building upon these operations, we conducted differential analysis and functional analysis between the two groups to better understand the underlying biological mechanisms, as described in** (Figure 1H)**. These biological functions were primarily enriched in coagulation, xenobiotic metabolism, bile acid metabolism, complement, fatty acid synthesis, peroxide synthesis, cholesterol homeostasis, fatty acid metabolism, and oxidative phosphorylation.

### 3.2 Construction and Validation of Risk Scores

**Figure 2:**
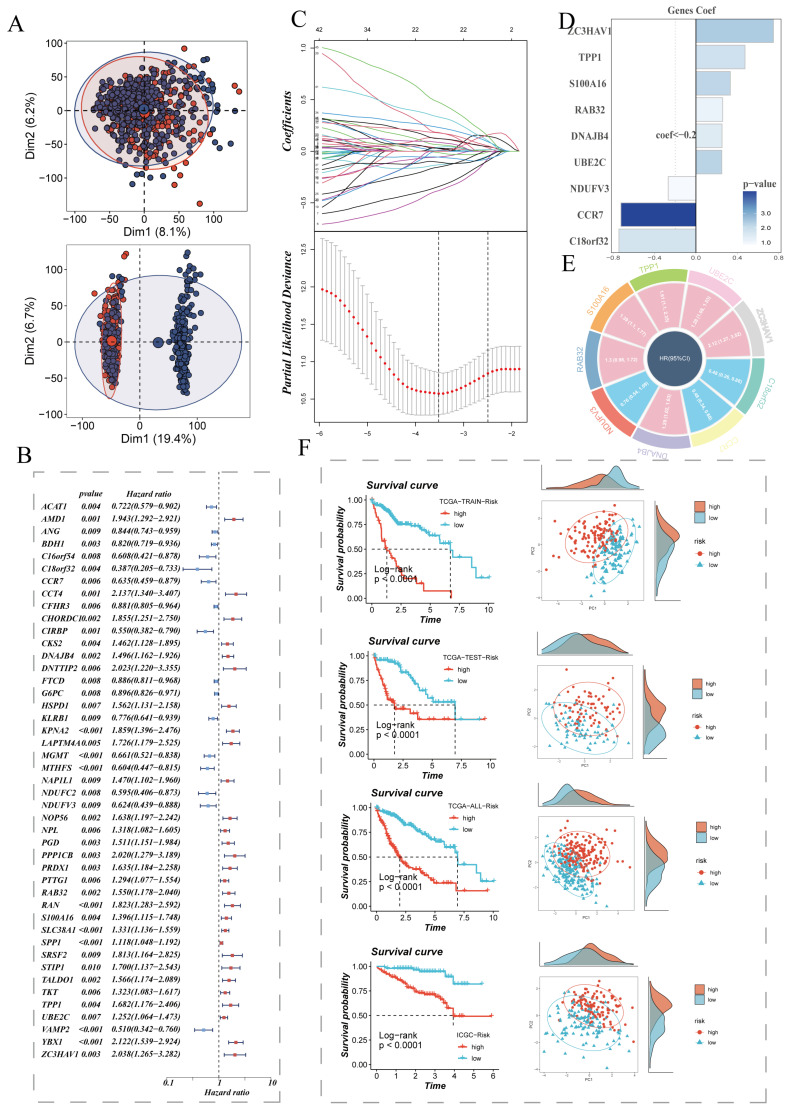
Establishment of the Sphingolipid Metabolism Signature and Key Gene Presentation. (A) PCA plots before and after batch effect removal in the liver cancer transcriptome dataset. (B) Forest plot showing prognosis-related genes. (C) LASSO regression analysis to select the most important model genes. (D, E) Coefficients of model genes and HR values of model genes. (F) Survival differences between high and low-risk groups.

We extracted differentially expressed genes between the high sphingolipid AUC group and low sphingolipid AUC group obtained from the above steps for bulk-level model construction. The sample distribution of TCGA data before and after batch effect removal is depicted in **(Figure 2A)**. On this basis, TCGA data samples were allocated to training and validation sets in a 6:4 ratio, and univariate Cox analysis was conducted, with results presented in a forest plot** (Figure 2B)**. The genes further screened were subjected to Lasso and multivariate Cox regression analysis** (Figure 2C)**, leading to the construction of a risk model composed of nine genes: C18orf32, CCR7, DNAJB4, NDUFV3, RAB32, S100A16, TPP1, UBE2C, ZC3HAV1. The coefficients of each model gene related to the risk score are shown in** (Figure 2E)**. The circos plot presents the respective HR values for the model genes, with NDUFV3, CCR7, C18orf32 as low-risk genes, and the remaining four as high-risk genes** (Figure 2E)**. Patients were further stratified into high-risk and low-risk groups based on the median risk value. Survival analysis showed statistically significant survival differences in the TCGA-HCC patient training set, test set, overall set, and the ICGC external validation dataset, demonstrating the prognostic model's robustness** (Figure 2F)**. PCA analysis based on the expression levels of model genes in TCGA and ICGC HCC samples indicated clear separation between high-risk and low-risk groups** (Figure 2F)**.

### 3.3 Establishment of a Nomogram for Model Evaluation

**Figure 3:**
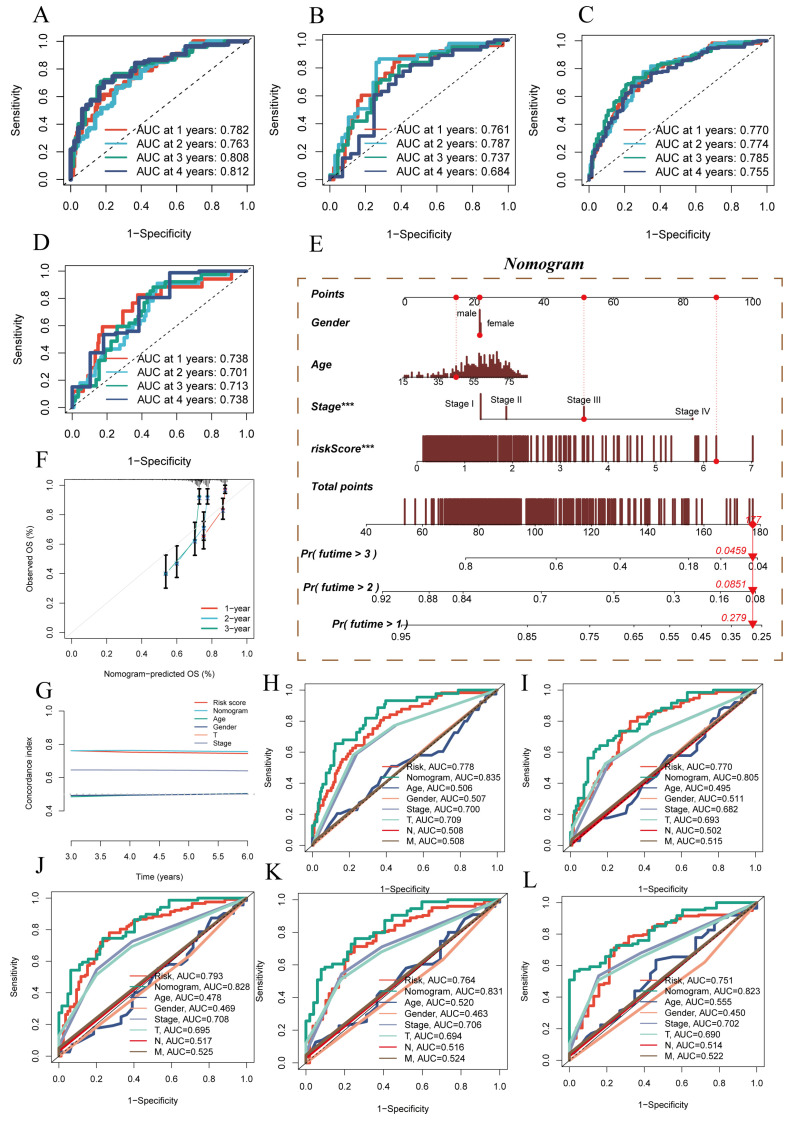
Model Evaluation and Nomogram Construction. (A-D) ROC curves showing diagnostic performance of the model in different groups. (E) Nomogram combining clinical features and risk scores. (F) Calibration plot testing consistency between actual OS rates and predicted survival rates, with the 45° line representing the best prediction. (G) C-index curve for evaluating predictive performance of different clinical features, nomogram scores, and risk scores. (H-L) ROC curves showing AUC values for various clinical factors, risk scores, and nomogram scores.

Additionally, ROC performance evaluation was used for further model validation. In the four datasets mentioned earlier, ROC performance was assessed for 1 year, 2 years, 3 years, and 4 years. The results, as shown in** (Figure 3A-D)**, demonstrated the superior performance of the prognostic model, with AUC values exceeding 0.7 in all the different datasets, although the ROC for the fourth year in the TCGA test set was 0.684. Based on the TCGA-HCC dataset, a predictive nomogram was constructed, incorporating risk scores and clinical-pathological parameters (age and clinical stage), to better predict prognosis** (Figure 3E)**. One-year, two-year, and three-year survival statuses were used as clinical outcome indicators. Calibration plots and the C-index curve indicated that the nomogram outperformed the risk score and any other clinical indicators in predicting prognosis** (Figure 3F)**. Moreover, we further employed ROC analysis to assess the predictive abilities of the nomogram score, risk score, and other clinical features. The ROC scores at different time points were all above 0.8 and surpassed other indicators included in the comparison** (Figure 3H-L)**.

### 3.4 Mutation Landscape Underlying Risk Model Stratification

**Figure 4:**
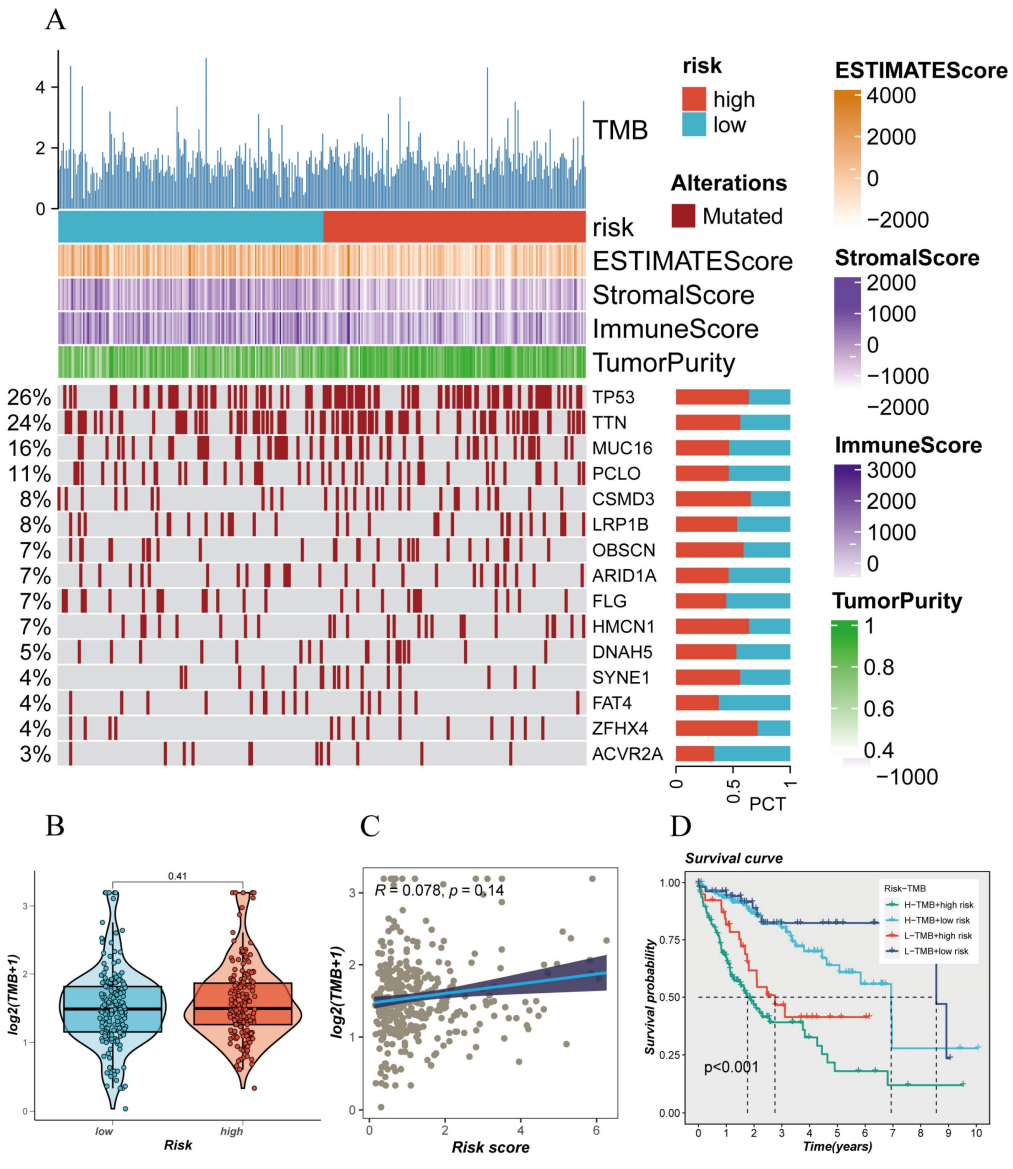
Mutation Spectrum of Liver Cancer Samples. (A) Mutation plot of top mutated genes in different risk subgroups. (B) Comparison of tumor mutation burden (TMB) between different risk groups. (C) Correlation analysis between risk scores and TMB. (D) Survival differences among four subgroups (H-TMB+ high-risk, H-TMB+ low-risk, L-TMB+ high-risk, and L-TMB+ low-risk).

The mutation status of the genome plays a crucial role in individualized cancer treatment, especially in immunotherapy. Analyzing the somatic gene mutation patterns in different risk populations revealed** (Figure 4A)** that the high-risk group had significantly higher mutation frequencies in genes such as TP53, TTN, CSMD3, LRP1B, HMCN1, SYNE1, ZFHX4, compared to the low-risk group. Moreover, this mutation frequency in the genome correlated with the trend of immune scores and tumor purity, suggesting a significant stratification of different populations in terms of immunotherapy response** (Figure 4A)**. Different risk groups also exhibited distinct tumor mutational burdens (TMB), with the high-risk group having a larger TMB** (Figure 4B)**. Furthermore, Spearman correlation analysis revealed a positive correlation between the risk score and TMB** (Figure 4C)**. To better dissect the relationship between the two, patients were divided into four groups based on the median TMB value and median risk value (H-TMB+H-R, HTMB+ L-R, L-TMB+ H-R, L-TMB+ L-R), showing that the L-TMB+ low-risk group had the best prognosis, while the H-TMB+ high-risk group had the worst prognosis** (Figure 4D)**, consistent with the previous analysis.

### 3.5 Immunological Features of Different Populations

**Figure 5:**
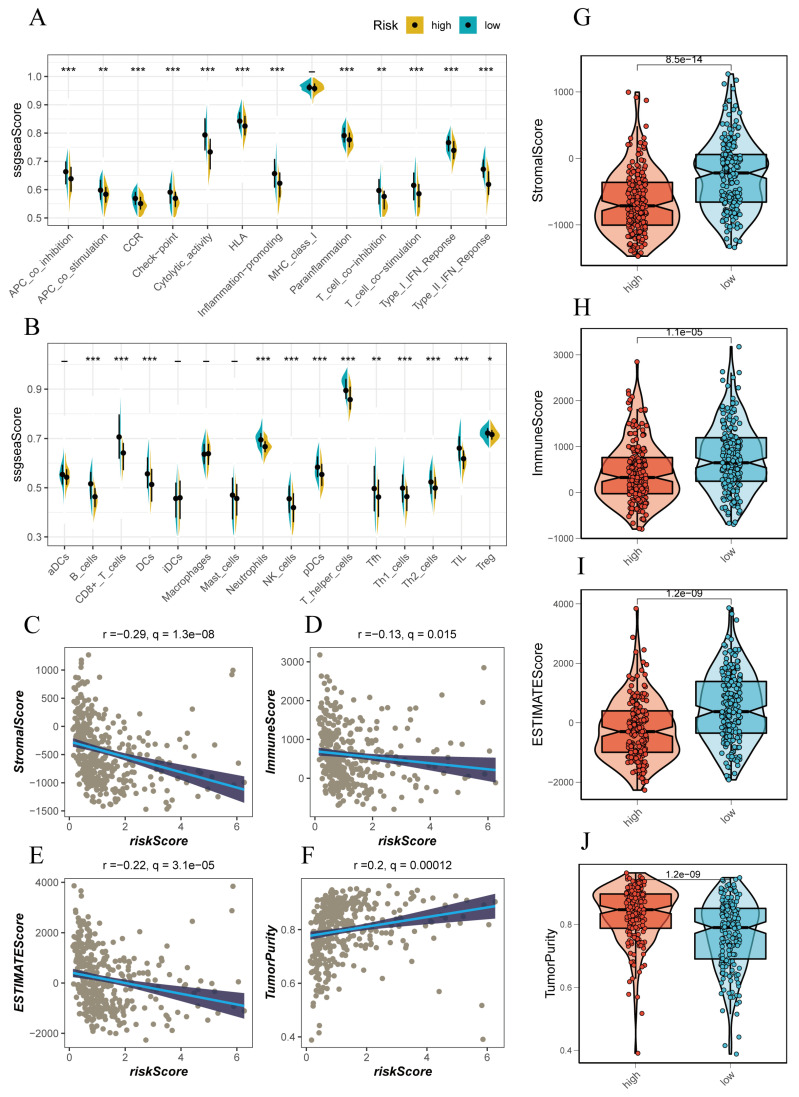
Immune Infiltration Characteristics Analysis. (A, B) ssGSEA algorithm evaluating immune infiltration differences between risk groups. (C-F) Correlation of stromal scores, immune scores, ESTIMATE scores, and tumor purity calculated using the ESTIMATE algorithm between the two risk subgroups. (G, J) Violin plots showing differences in stromal scores, immune scores, ESTIMATE scores, and tumor purity calculated using the ESTIMATE algorithm between the two risk subgroups.

Using the ssGSEA method, we conducted an immunoinfiltration assessment of high-risk and low-risk groups. The high-risk group tended to have higher scores in APC-co-inhibition, checkpoints, cytolytic activity, MCH-1 class, T cell co-inhibition, and IFN response, indicating a more intense immune response in this group** (Figure 5A, B)**. Further assessment of this immune response revealed that the high-risk group was accompanied by more immune cell infiltrations, such as T cells, B cells, NK cells, and activated macrophages** (Figure 5A, B)**. We attempted to evaluate the relationship between the risk score and the immune infiltration score using Spearman correlation analysis. The risk score was negatively correlated with Stromal score (R=-0.29), Immune score (R=-0.13), ESTIMATE score (R=-0.22), and positively correlated with tumor purity (R=0.2)** (Figure 5C-F)**. This correlated with the trend of the above scores in high-risk and low-risk groups, where the low-risk group had lower Stromal score, Immune score, and ESTIMATE score compared to the high-risk group** (Figure 5G-J)**.

### 3.6 Prognostic Model Prediction of HCC Patients' Response to ICIs

**Figure 6:**
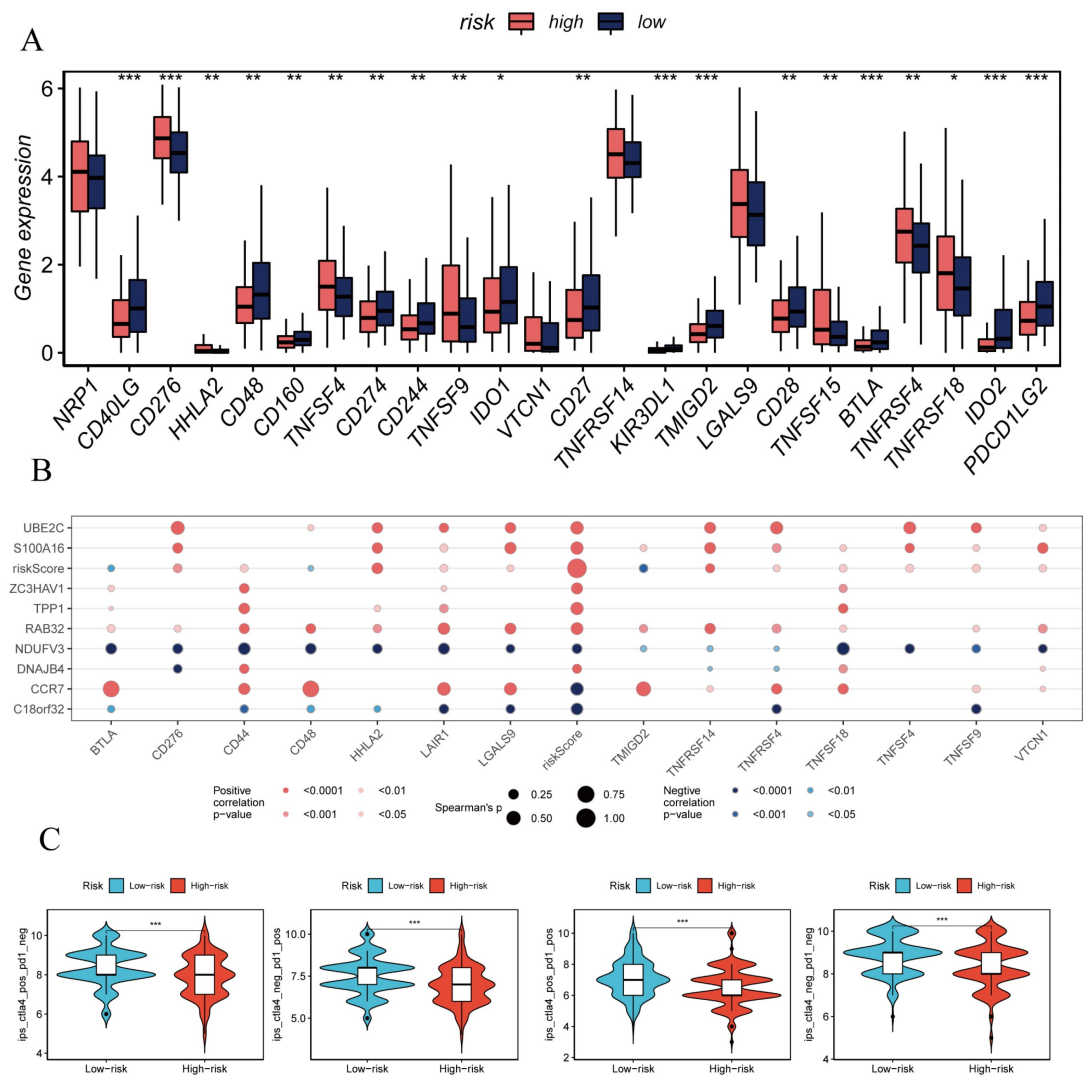
Immune Checkpoint Analysis. (A) Box plot showing differences in immune checkpoint gene expression between high-risk and low-risk groups. (B) Correlation between model genes and immune checkpoints. (C-F) IPS, IPS-CTLA4-neg-PD-1neg, IPS-CTLA4-pos-PD-1-neg, IPS-CTLA4-neg-PD-1-pos, and IPS-CTLA4-pos-PD-1-pos significantly increased in the low-risk group.

**Figure 7:**
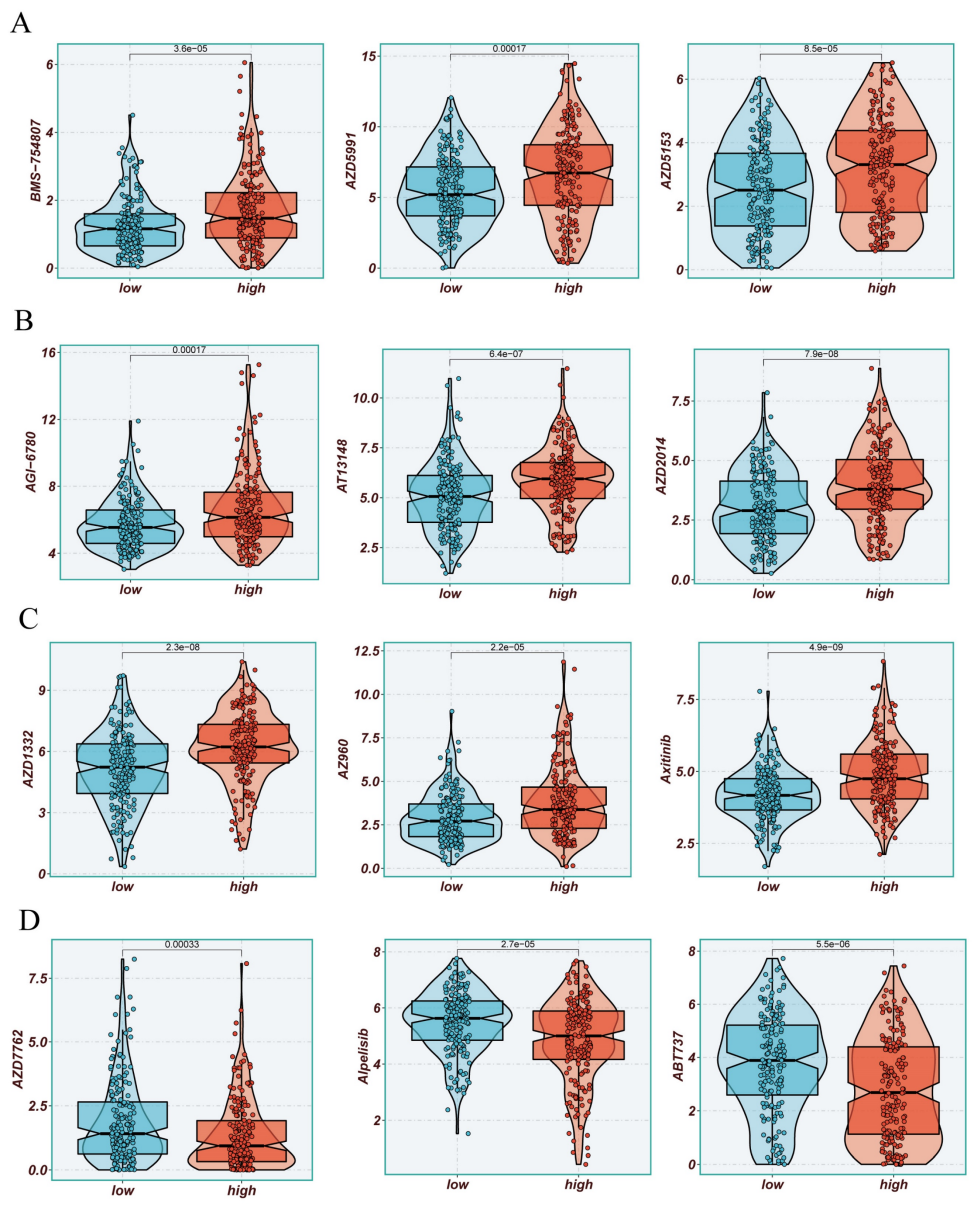
Drug Sensitivity Analysis. (A-D) Sensitivity to common cancer treatment drugs in high and low-risk groups.

The previously established fact that the risk score is correlated with the degree of immune cell infiltration and the quantity of various components in the TME leads to the investigation of whether the prognostic model can predict the response of HCC patients to immune checkpoint inhibitors (ICIs). Initially, we explored the relationship between the well-accepted ICI biomarkers and Riskscore, revealing interestingly inconsistent correlations. For instance, the overall expression levels of CD276, NRP1, TNFSF9, LGALS9 were higher in the high-risk group, while CD40LG, CD48, IDO1, and other biomarkers were more dominant in the low-risk group** (Figure 6A, B)**. IPS analysis could assist in more accurately identifying individuals benefiting from immunotherapy, thus achieving precision treatment. Assuming positive immune responses for IPS, IPS-CTLA4-neg-pd-1neg, IPS-CTLA4-pos-pd-1-neg, IPS-CTLA4-neg-pd-1-pos, and IPS-CTLA4-pos-pd-1-pos, the IPS score for the low-risk group was significantly higher, indicating that they would benefit the most from this form of immunotherapy** (Figure 6C)**. Consequently, we further screened potentially friendly drugs for different populations. Overall, the advantageous drugs for different risk groups varied, possibly depending on the heterogeneity of different populations, with drugs like Axitinib, AZD1332, AZ960, AT13148 being more favorable for the high-risk group** (Figure 7A-D)**. Utilizing seven different immune assessment algorithms also indicated that tumors in the low-risk group had more immune cell infiltration, as shown in the heatmap of macrophages, T cells, NK cells, etc. **(Figure 7A-D)**.

### 3.7 Enrichment Analysis Explaining Characteristics of Different Populations

We attempted to analyze HALLMARK pathway gene signatures to reveal distinct features between high-risk and low-risk groups. Patients in the high-risk group exhibited enrichment in biological mechanisms such as MYC_TARGETS, G2M CHECKPOINT, WNT_BETA_CATENIN_SIGNALING, while those in the low-risk group were more inclined towards FATTY_ACID_METABOLISM, XENOBIOTIC_METABOLISM, ADIPOGENESIS, and other biological mechanisms **Supplementary Figure 1B)**.

### 3.8 Validation of Key Genes in the Model

**Figure 8:**
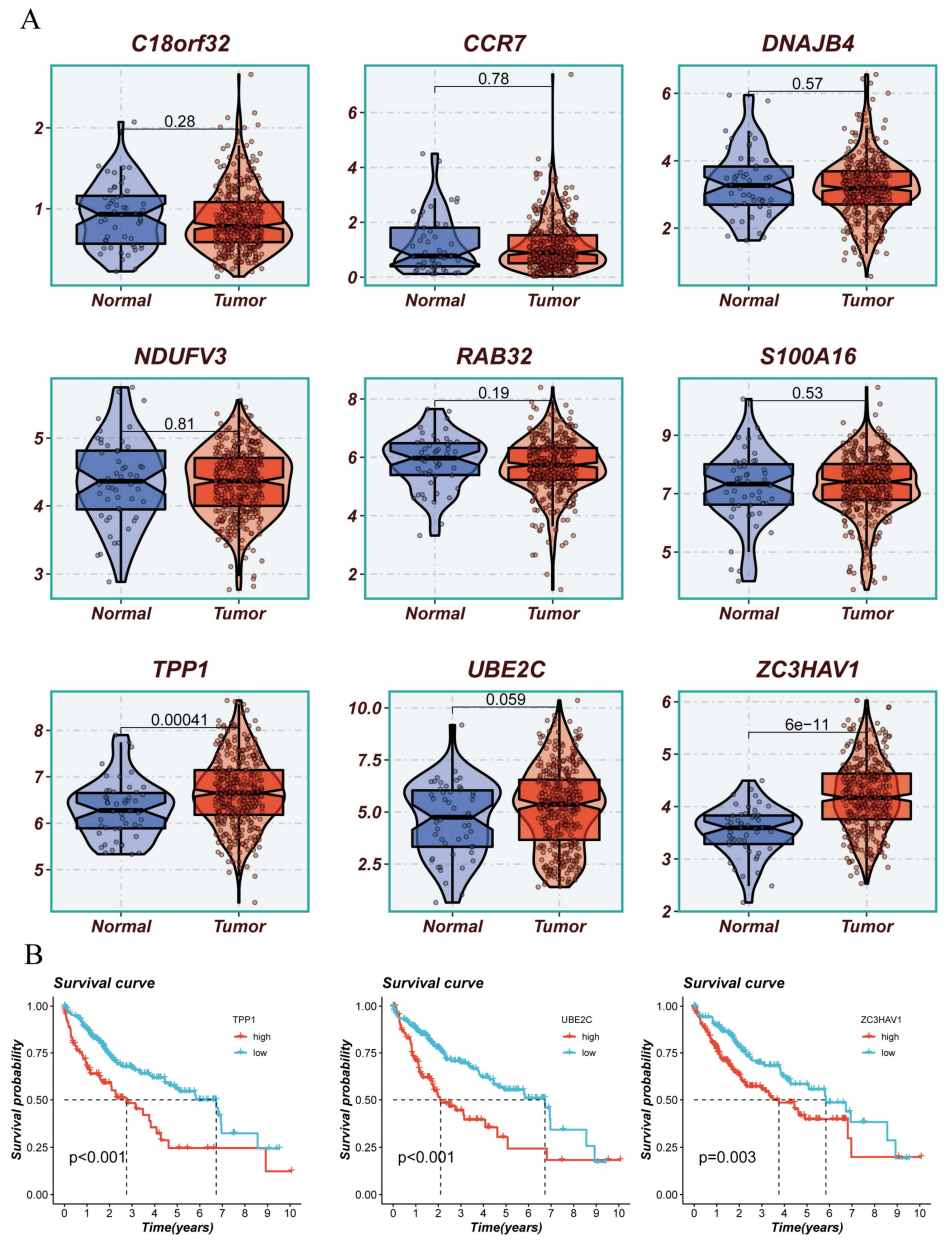
Presentation of Model Constructing Genes and Survival Analysis of Representative Genes. (A) Differences in expression of model constructing genes between tumor and normal tissues. (B) Survival analysis of differentially expressed genes.

**Figure 9:**
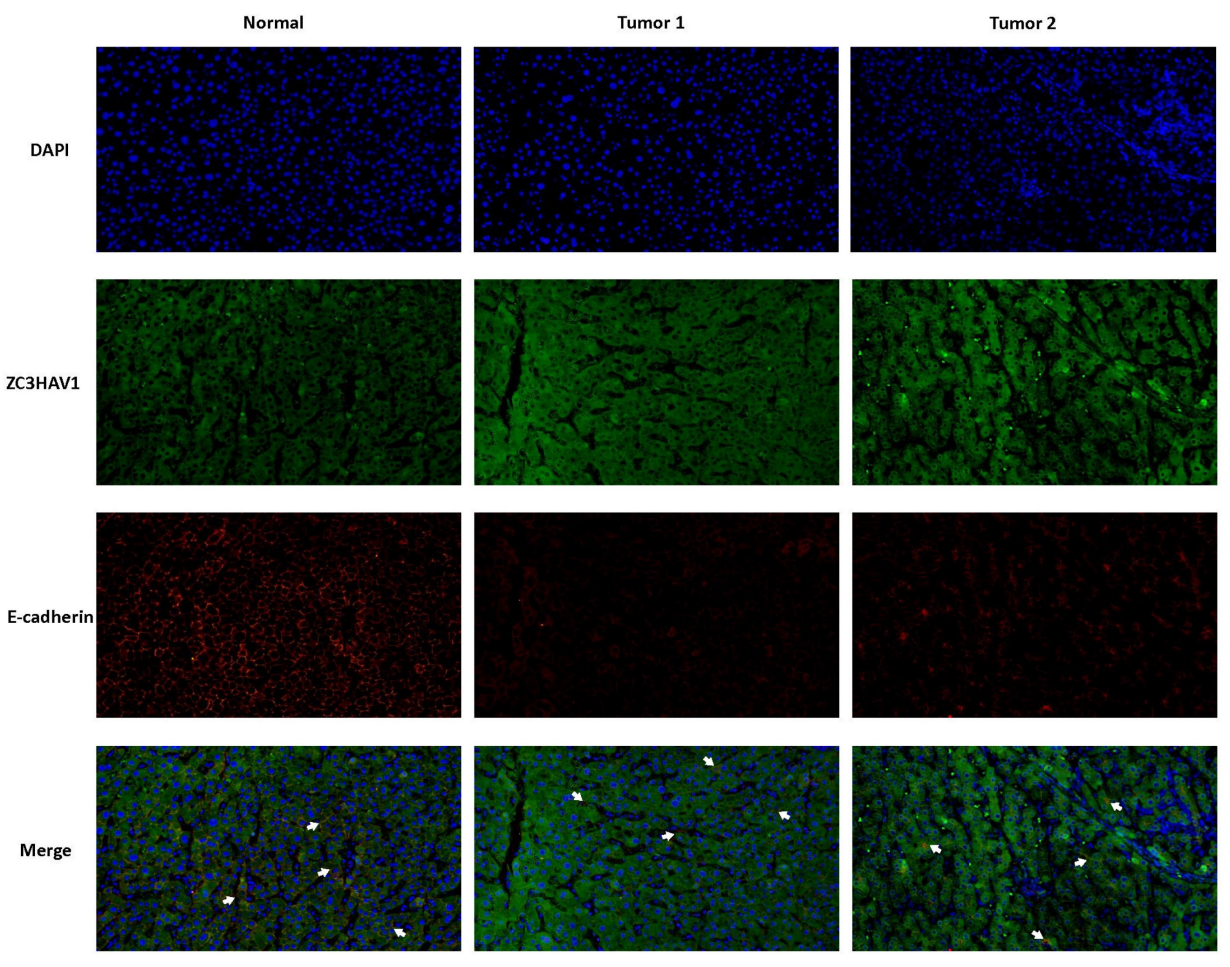
Multiplex Tissue Immunofluorescence Explaining the Relationship between Key Genes and EMT.

The above analysis confirmed that the Risk Score can effectively stratify HCC, aiding future precision treatments. This characteristic of the Risk Score largely stems from the efficacy of model genes. Therefore, we conducted KM survival analysis on the nine model genes, with results shown in** (Figure 8A, B)**. Three genes, TPP1, UBE2C, ZC3HAV1, were significantly associated with the survival information of HCC patients. Given the relative gap in research on ZC3HAV1 in HCC, we conducted multiplex immunofluorescence to preliminarily explore its biological functions. The results showed differential expression levels of ZC3HAV1 between cancer and non-cancer samples, specifically, significantly higher expression in HCC samples compared to healthy samples. Additionally, we evaluated the relationship between ZC3HAV1 and a crucial EMT indicator, E-cadherin. E-cadherin is a protein that plays a crucial role in cell-cell adhesion, typically highly expressed in epithelial cells, maintaining tight connections between cells. The results indicated that when ZC3HAV1 expression was upregulated, E-cadherin expression decreased, suggesting that ZC3HAV1 might influence the malignant progression of HCC patients by affecting the EMT process** (Figure 9**.

## 4. Discussion

The therapeutic strategies for hepatocellular carcinoma (HCC), a refractory malignancy characterized by multifactorial and intricate molecular mechanisms, primarily hinge upon the stage of the lesion and the early diagnosis is pivotal for enhancing treatment efficacy^25^, ^26^. Approaches vary based on the interplay between surgical resection and the application of combinatory drug treatments. A favorable aspect is the ongoing progress in drug therapy research, with a focus on specific targeted drugs and immunotherapy gaining prominence in both research and clinical practice^27^. Nevertheless, challenges persist, including difficulties in early diagnosis, the development of drug resistance, and the complexity of pathological molecular mechanisms. The future exploration of more effective treatment strategies and the potential for individualized therapy is paramount. For the majority of advanced-stage HCC patients, surgery and radiation therapy may not sufficiently salvage the less optimistic survival rates. Tumor metastasis is a result of the concerted action of specific genes and signaling pathways. Disrupting any step in this process may impede the formation of metastases^28^.

Immunotherapy, by weakening the pro-tumor effects or enhancing anti-tumor effects of the body's immune system, can more effectively treat HCC patients. Targeting immune cells in the specific immune microenvironment of HCC induces a diverse array of functions, making the immune microenvironment a potent target for cancer treatment 27, 28. The development of single-cell RNA sequencing (scRNA-seq), characterized by the ability to achieve fair and high-resolution analyses of intracellular mechanisms, may aid in understanding the cellular transformations and intricate roles of immune cells in the HCC immune microenvironment 29, 30.

As previously mentioned, our study integrates current single-cell and transcriptome technologies to develop a model capable of effectively assessing the characteristics of the HCC immune microenvironment and predicting the survival prognosis of HCC patients. The model comprises nine genes frequently encountered in current tumor research. CCR7, a G protein-coupled receptor typically expressed on lymphocytes, plays a crucial role in regulating the migration of immune cells to lymph nodes. The CCR7 axis, involving the ligands CCL19 and CCL21, may play a dual role in the growth, invasion, metastasis, and immune escape of tumors 31. It mediates tumor expansion by activating intracellular PI3/akt, MAPK/ERK, and Jak/STAT signaling pathways. However, the CCR7 axis significantly contributes to coordinating immune responses, especially in promoting the recognition between antigen-presenting dendritic cells and antigen-responsive naïve lymphocytes, thus counteracting tumor expansion 32, 33. RAB32, a member of the RAS protein family, correlates with the malignancy and poor prognosis of tumors. It has been confirmed to promote the proliferation and invasive capabilities of GBM cells by activating the ERK signaling pathway 34. DNAJB4, associated with the severity and poor prognosis of triple-negative breast cancer, promotes apoptosis in these cancer cells by activating the Hippo signaling pathway. This mechanism involves regulating the expression of YAP1 (Yes-associated protein 1), influencing cell proliferation and invasion capabilities, providing a novel target for cancer treatment 35. C18orf32, an open reading frame (ORF) gene, facilitates tumor growth and dissemination by influencing the PI3K/Akt signaling pathway, altering the cell cycle process 36. Elevated expression of S100A16 in the tumor microenvironment promotes the formation of tumor-related blood vessels, facilitating tumor growth 37. UBE2C promotes cancer cell proliferation by activating the AKT/mTOR signaling pathway and serves as a novel target in lung cancer associated with Kras mutations 38. Additionally, TPP1 is a component of the shelterin protein complex that protects chromosome ends from unnecessary end-to-end fusions, contributing to maintaining telomere stability. It can reduce the sensitivity of esophageal cancer to cisplatin chemotherapy and, when highly expressed, can promote tumor cell proliferation and migration while also chemosensitizing DNA damage and downregulating protein expression through the ATM/ATR-p53 signaling pathway 39. Our subsequent focus on ZC3HAV1, a member of the PARP protein family, known as poly (ADP-ribose) polymerase-13 (PARP13), indicates its potential roles in regulating specific mRNA stability and translation as an RNA-binding protein. It can also modulate the miRNA silencing pathway, comprehensively impacting miRNA targets 40.

Research suggests that ZC3HAV1, on one hand, upregulates the expression of cyclin D1 and cyclin-dependent kinase 2 in digestive tract tumor cells, promoting the G1/S transition of digestive tract tumor cells. It also upregulates the expression of epithelial-mesenchymal transition (EMT)-related markers 41. Furthermore, it can directly bind to KRAS and positively regulate its expression. Persistent aberrant expression of KRAS directly activates the MAPK signaling pathway. Overexpression of ZC3HAV1 also activates the MAPK signaling pathway by increasing p-ERK levels, mediating tumor proliferation, apoptosis, invasion, and migration 41, 42. On the other hand, PARP13 restricts oncogenic viruses, inhibits the expression of the pro-survival cytokine receptor TRAILR 4, and prevents malignant transformation and cancer development. Thus, it mediates tumor proliferation, apoptosis, invasion, and migration 43. However, the current research on ZC3HAV1 in liver cancer remains limited, and preliminary experiments in liver cancer tissues suggest an elevated expression of this gene. We speculate and validate its potential association with EMT, as mentioned earlier.

Previous studies have highlighted the role of sphingolipid metabolism across various cancer types, with researchers employing diverse bioinformatics tools to explore its potential applications 44, 45. Our study, for the first time in the field of liver cancer, applies the latest single-cell technology to evaluate this. As mentioned earlier, we grouped TCGA patients based on the established risk score, and different validation cohorts consistently showed that high-risk patients correspond to low survival rates. Subsequent integration of clinical information was carried out to develop a line chart, which exhibits superior performance in predicting survival compared to risk scores and other clinical features. Currently, our attention is directed towards understanding the distinct characteristics of high and low-risk populations. Gene mutation information is a critical aspect of our research, and a significant feature in the high-risk population is the higher frequency of TP53 mutations. Notably, the high-risk group is marked by a higher frequency of TP53 mutations, offering a plausible explanation for their compromised tumor suppressor function and resistance to chemotherapy and radiation 46.

Additionally, the level of tumor mutational burden (TMB) is associated with the genetic variation burden of tumors. High TMB may indicate the presence of more mutations in tumor cells, making them more easily recognizable and attackable by the immune system 47, 48. Although our study found no significant differences in TMB scores between high and low-risk populations, we further divided patients based on TMB and risk status into four groups. Among these, the H-TMB+ low-risk group exhibited a better prognosis, presenting opportunities for further exploration. To gain a deeper understanding of how the tumor microenvironment (TME) affects tumor prognosis, we examined the immune characteristics of high-risk and low-risk HCC patients. Applying the ESTIMATE method for an overall sample immune information assessment, the results indicated a negative correlation between the risk score and stromal, immune, and ESTIMATE scores. This may provide decision-making insights in clinical settings when facing neoadjuvant therapy. As mentioned in our subsequent research analysis, suggesting the use of certain widely used drugs such as Axitinib, AZD1332, AZ960, AT13148 based on sphingolipid metabolism, we offer recommendations for high and low-risk populations, potentially assisting in improving the overall survival of HCC patients.

In conclusion, although our current study is limited to cross-validation of multicenter datasets and in-depth laboratory mechanism exploration, we have at least demonstrated that a model constructed with nine SRGs can effectively predict the prognosis of HCC patients and provide potential directions for guiding future HCC treatments.

## Supplementary Material

Supplementary figure.
