# Supplementary material for: Immunofluorescence-Verified Sphingolipid Signatures Indicate Improved Prognosis in Liver Cancer Patients
Source: J Cancer. 2024 Oct 14;15(19):6239–55. doi: 10.7150/jca.101330 (PMC11540515; doi:10.7150/jca.101330)

Supplementary Figure 1. (A) Immune infiltration differences between risk groups evaluated by seven algorithms. (B) Gene set enrichment analysis of patients in different risk groups.

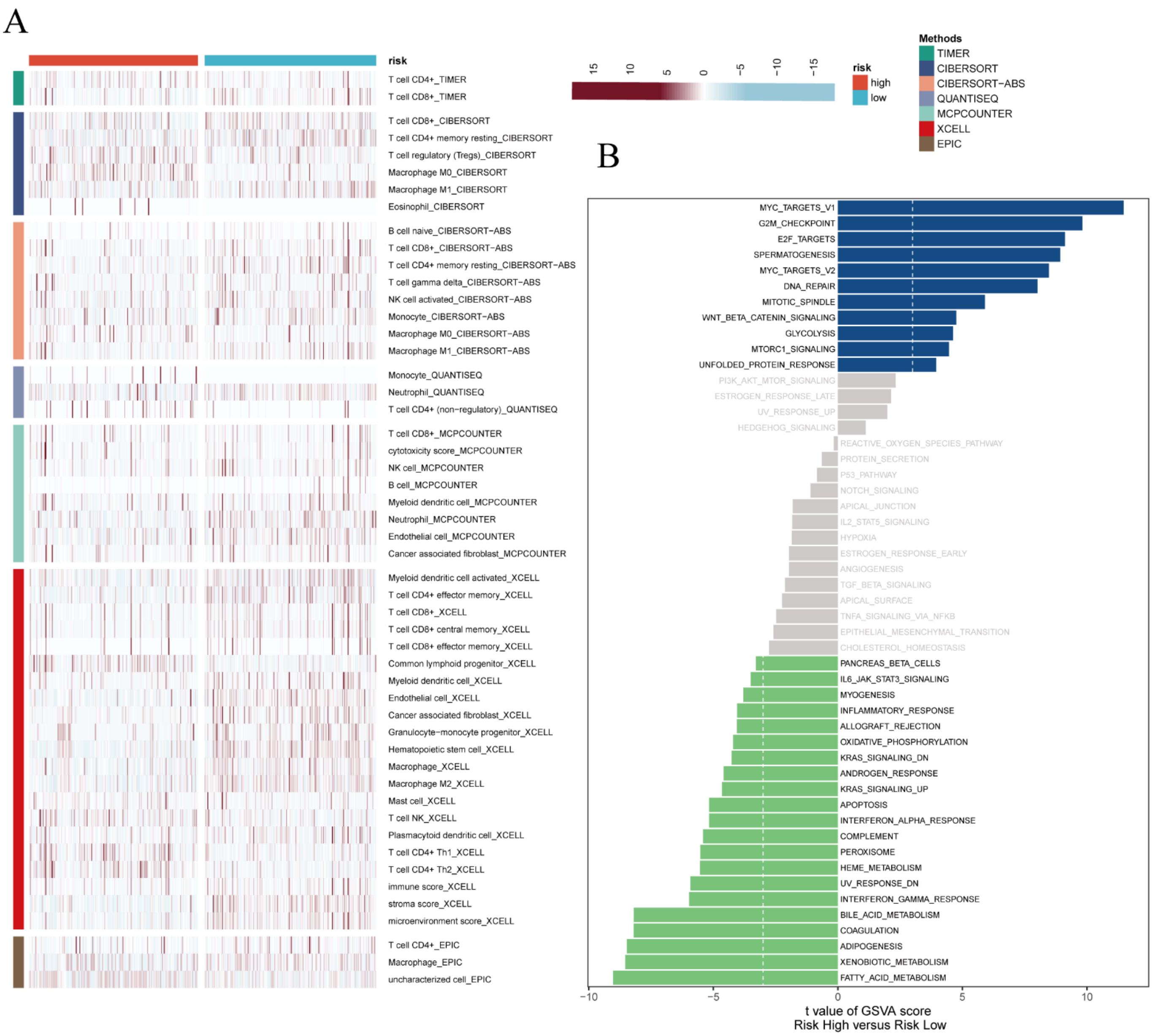

Supplement: Supplementary file 1 — Supplementary figure. [file jcav15p6239s1.pdf]
